# Tuning Molar Mass of the D18 Polymer via Stille Polymerization: Impact on Morphology and Large-Area Blade-Coated Organic Solar Cells

**DOI:** 10.1021/acsomega.5c12462

**Published:** 2026-03-02

**Authors:** Renata S. Cardoso, Igor T. Soares, João A. F. L. Batalha, Isabela C. Mota, Lucas G. P. Tienne, Tamires Y. G. Alves, Letícia A. Marcate, Juliana L. S. Martins, Gabriela A. Soares, Bárbara H. S. Miranda, Diego Bagnis, Erica G. Chaves, Maria de Fátima V. Marques

**Affiliations:** 1 Instituto de Macromoléculas Professora Eloisa Mano (IMA), 28125 Universidade Federal do Rio de Janeiro, Av. Horácio Macedo, 2030, CT-Bloco J, Rio de Janeiro, RJ 21941-598, Brazil; 2 ONINN Centro de Inovações, Avenida José Cândido da Silveira, 2000, Horto Florestal, Belo Horizonte, MG 31035-536, Brazil; 3 Centro de Pesquisas, Desenvolvimento e Inovação Leopoldo Américo Miguez de Mello (Cenpes, PETROBRAS), Av. Horácio Macedo, 950, Rio de Janeiro, RJ 21941-915, Brazil

## Abstract

Achieving reproducible, high-performance organic solar cells (OSCs) requires precise control over the molar mass of donor polymers, as it governs film formation, morphology, and charge transport. Here, we systematically investigate the influence of Stille polymerization conditions on the molar mass, optoelectronic properties, morphology, and device performance of the benchmark donor polymer D18. By varying reaction time, catalyst type, and catalyst loading, we access D18 batches with weight-average molar masses (*M*_w_) ranging from approximately 12 to 93 kg·mol^–1^. Gel permeation chromatography, UV–vis absorption, cyclic voltammetry, optical microscopy, profilometry, and AFM indicate that higher-*M*_w_ polymers exhibit enhanced aggregation signatures, as well as smoother, more compact films, and improved donor–acceptor phase separation compared to low-*M*_w_ analogues. Bulk heterojunction OSCs with an inverted architecture (R2R-patterned PET/IMI)/ZnO/D18:Y6:PC_70_BM/PEDOT:PSS/Ag) are fabricated by blade coating under ambient atmosphere over large active areas (0.55 cm^2^). Devices based on low-*M*_w_ D18 (*M*_w_ ≈ 12–14 kg·mol^–1^) show poor performance (PCE < 2%), which correlates with low shunt resistance and unfavorable morphology, whereas high-*M*_w_ D18 samples (*M*_w_ ≈ 83–93 kg·mol^–1^) reach power conversion efficiencies of 7.8–8.0%, approaching that of a commercial D18 reference (8.9%). A solvent study further reveals that halogenated solvents (chloroform, chlorobenzene) are required to fully realize the potential of high-*M*_w_ D18, while *o*-xylene yields more homogeneous films and competitive efficiencies primarily for low-*M*_w_ material. These findings highlight molar mass control and solvent selection as interdependent parameters for optimizing morphology and device performance in scalable, blade-coated D18-based organic solar cells.

## Graphical abstract

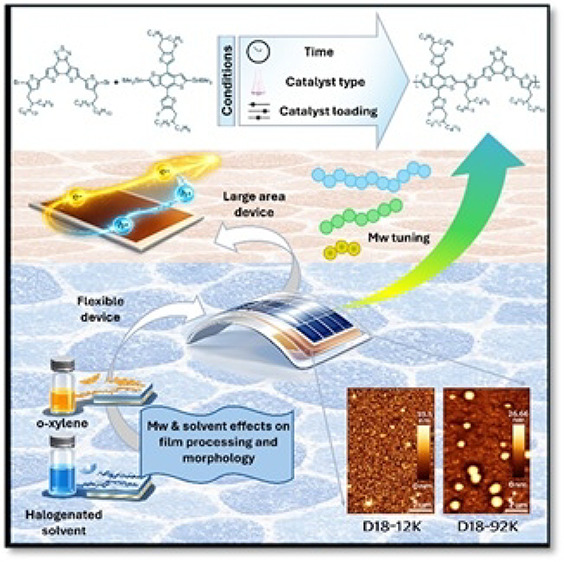

## 1 Introduction

High-performance semiconducting polymers have been central to the rapid development of organic solar cells (OSCs), in which the chemical structure, intermolecular organization, and molar mass of the donor polymer are key parameters influencing device efficiency. Among the new-generation donor materials, D18 has emerged as a benchmark polymer due to its narrow bandgap, strong absorption in the visible region, high hole mobility, and excellent compatibility with state-of-the-art nonfullerene acceptors (NFAs).^1,2^ These characteristics have positioned D18 as a reference system for studying structure–property–performance relationships in OSCs.

Structurally, D18 contains alternating benzodithiophene (BDT) donor units and fused dithienobenzothiadiazole (DTBT) acceptor units, which promote a relatively coplanar backbone and favorable intermolecular interactions, supporting efficient π–π stacking and phase separation in bulk-heterojunction (BHJ) blends.^3^ As a result, optimized D18-based devices have achieved power conversion efficiencies exceeding 19%, including a *J*_sc_ of 26.86 mA·cm^–2^ and FF of 77.25% in combination with L8-BO.^3^ It is important to note that these record efficiencies were obtained under ideal fabrication conditions, namely in a controlled inert atmosphere (glovebox) and using devices with small active areas.

Beyond binary blends, D18 has also been explored as a morphology regulator in ternary systems, improving crystallinity and tune phase separation, enabling enhanced open-circuit voltage (*V*_oc_) values associated with its relatively deep HOMO level.^4–6^ Related derivatives and synthetic variants, such as D18-Cl, further illustrate how subtle changes in polymer structure and molecular characteristics can impact device performance, with efficiencies up to 16.6% reported for *M*_w_ ≈ 56 kg·mol^–1^.^7^ Despite these advances, a persistent challenge in the literature is the batch-to-batch variability commonly observed for donor polymers synthesized via Stille coupling, in which polymerization time, catalyst type, monomer purity, and concentration can strongly influence the resulting molar mass and dispersity.

Polymer molar mass plays a critical but nontrivial role in determining film-forming ability, chain packing, crystallinity, domain size, phase purity, and ultimately charge transport within the BHJ.^8^ Low *M*_w_ polymers can suffer from insufficient chain entanglement and poor film cohesion, leading to limited percolation pathways and low shunt losses, whereas very high *M*_w_ may exhibit reduced solubility and narrower processing windows, potentially compromising film uniformity and BHJ miscibility. *M*_w_ further influences blend thermodynamics and film-formation kinetics by modulating chain mobility, crystallization rates, and phase-separation dynamics during solvent evaporation.^9^ Consequently, optimal photovoltaic performance is often achieved only within a limited *M*_w_ range, highlighting the importance of precise synthetic control for both reproducibility and scalability.

While record-efficiency devices are often fabricated via spin coating over small areas (<0.1 cm^2^), scalable deposition techniques, such as blade coating, slot-die coating, and roll-to-roll (R2R) printing are required for industrial implementation.^10,11^ Blade coating offers a versatile platform for scalable film deposition, allowing control over thickness and uniformity through parameters such as blade speed, substrate temperature, and ink concentration. However, scaling from laboratory to large-area processing introduces additional complexity, including altered drying dynamics, prolonged exposure to air, film thickness, and thickness variations during layer printing.^12^ In this context, the influence of polymer molar mass becomes particularly important, as chain entanglement, aggregation behavior, and solution rheology directly affect film formation during blade coating.

Despite the extensive use of D18 in high-efficiency OSCs, systematic studies that directly correlate Stille polymerization conditions, molar mass distribution, film morphology, and device performance remain limited, especially for large-area devices fabricated under ambient conditions using scalable coating techniques. This gap complicates reproducibility across laboratories and hinders rational process optimization for manufacturing-relevant conditions.

In the present study, we synthesize multiple D18 batches by varying Stille polymerization conditions, including reaction time, catalyst loading, and catalyst type, thereby accessing polymers spanning a wide *M*_w_ range. We systematically correlate *M*_w_-dependent trends in optical properties, film morphology, and photovoltaic performance in BHJ devices fabricated by blade coating over large active areas (0.55 cm^2^). Rather than proposing universal mechanistic models, our results provide experimentally grounded insights into how molar mass and processing conditions jointly govern morphology and device performance, underscoring the importance of synthetic control for scalable OSC manufacturing.

## 2 Materials and Methods

### 2.1 Materials

The monomers 5,8-bis­(5-bromo-4-(2-butyloctyl)­thiophen-2-yl)­dithieno­[3′,2’:3,4;2’’,3′’:5,6]­benzo­[1,2-*c*]­[1,2,5]­thiadiazole (named D18–2Br, 98%) and 2,6-bis­(trimethyltin)-4,8-bis­(5-(2-ethylhexyl)-4-fluorothiophen-2-yl)­benzo­[1,2-b:4,5-b’]­dithiophene (BDTTDFSn, 98%) were purchased from Alfa Chemical Co., Ltd. (China) and used without further purification. The palladium catalyst tris­(dibenzylideneacetone)­dipalladium(0) (Pd_2_(dba)_3_, stated purity ≥ 98%) and the ligand tri­(o-tolyl)­phosphine, (P­(o-tol)_3_, stated purity ≥ 98%) were obtained from Sigma-Aldrich, Brazil. Commercial D18 (denoted as D18-C; *M*_w_ = 91,999 g·mol^–1^; *M*_n_ = 40,877 g·mol^–1^, as provided by the supplier) was acquired from Ossila Ltd., UK, and used as a reference material without further purification.

All air- and moisture-sensitive reagents were handled under an inert nitrogen atmosphere using standard Schlenk techniques. Toluene (Sigma-Aldrich, Brazil) was dried over molecular sieves and freshly distilled over sodium/benzophenone prior to use. Methanol, dichloromethane, chloroform, and chlorobenzene (Merck KGaA, Brazil, analytical grade) were used as received without further purification.

Flexible roll-to-roll patterned PET/ITO/Ag/ITO substrates supplied by OIKE & Co., Ltd. (Japan) were used. These substrates consist of poly­(ethylene terephthalate) coated with an ITO/metal/ITO (IMI) transparent electrode and are hereafter referred to as PET/IMI.

Poly­(3,4-ethylenedioxythiophene):poly­(styrenesulfonate) (PEDOT:PSS, AI 4083 grade) was purchased from Ossila Ltd. and used as received.

### 2.2 Polymer Synthesis and Characterizations

D18 polymers were synthesized via Stille cross-coupling polymerization. In a nitrogen-purged Schlenk flask, the monomers D18–2Br and BDTTDFSn were dissolved in anhydrous toluene and connected to a reflux condenser. The reaction mixture was degassed by three freeze–pump–thaw cycles to ensure an inert atmosphere. Once the reaction mixture reached 110 °C, solutions of palladium catalyst (Pd_2_(dba)_3_ or Pd­(PPh_3_)_4_, depending on the experiment) and the corresponding phosphine ligand (P­(o-tol)_3_, when applicable) were introduced. Polymerization was conducted under reflux for different reaction times, catalyst loadings, and palladium sources, as summarized in Table 1.

**Table 1:** Polymerization Conditions and GPC Results of D18 Copolymers^a^

		catalyst		GPC results		
polymer donor	polym.Time (h)	Pd_2_(dba)_3_/ P(o-tol)_3_(1:10) (mol %)	Pd(PPh_3_)_4_(mol %)	*M*_n_(kg mol^–1^)	*M*_w_(kg mol^–1^)	Đ
^a^D18–12K	12	1.50		6.66	12.22	1.83
^b^D18–14K	24	1.50		6.64	14.46	2.18
^c^D18–23K	24		1.50	12.38	23.71	1.92
^d^D18–92K*	24	1.13		44.76	92.79	2.18
^e^D18–83K	16	1.50		42.65	83.66	1.98

a Reaction conditions: toluene as solvent; reaction temperature of 110 °C. Polymers a–d were synthesized at a monomer concentration of 0.083 mmol mL^–1^, whereas polymer ^e^D18–83K was synthesized at a higher monomer concentration of 1.0 mmol mL^–1^. ^d^D18–92K* was synthesized with lower catalyst load. For comparison, the commercial polymer D18C-92K has *M*_n_ = 40,877 g·mol^–1^ and *M*_w_ = 91,999 g·mol^–1^ (Đ ∼ 2.25), values provided by the supplier.

After completion, the mixture was cooled to 60 °C and diluted with an additional 50 vol % of toluene. Following 1 h of stirring, the reaction mixture was precipitated dropwise into cold methanol under vigorous stirring. The crude polymers were purified by Soxhlet extraction using successively dichloromethane/chloroform (1:1, v/v), chloroform and chlorobenzene.^1,13^ This purification step was employed to remove residual monomers, catalyst residues, and low-molar-mass oligomeric fractions, while preserving the main polymer fraction. The selected solvent ratio provides sufficient solvency for small molecular species while limiting dissolution of higher-molar-mass D18 chains, thereby minimizing undesired polymer loss during purification.

Gel permeation chromatography (GPC) was performed using an Agilent 1260 Infinity II high-temperature system equipped with a refractive index detector. Separation was carried out with an Agilent PLgel 10 μm Mixed-B 300 × 7.5 mm column (PN: PL1110–6100) with 1,2,4-trichlorobenzene as the eluent at 160 °C. Weight-average molar mass (*M*_w_), number-average molar mass (*M*_n_), and dispersity (Đ) were determined by calibration with narrow polystyrene standards.

Atomic force microscopy (AFM) was carried out using a JPK Nanowizard (Bruker Co., USA), using a silicon cantilever with a spring constant of 3 N.m^–1^. The samples were prepared by depositing the active layer on a PET/IMI flexible substrate previously covered with a ZnO layer, using the same device fabrication conditions and chloroform as the solvent. AFM system (tapping mode) to investigate the surface morphology of the films.

Surface roughness values were independently quantified by stylus profilometry using a DektakXT profilometer (Bruker Co., USA), with five 2 mm line scans collected at different locations on each sample. The analyzed samples consisted of electron transport layer (ETL) and active layers deposited sequentially. The active layers used for AFM were prepared using the same solvent and additive conditions employed for the corresponding device sets (chloroform, chlorobenzene, or *o*-xylene, with additive usage as specified in the tables).

UV–vis absorption spectra were recorded using a Thermo Scientific BioMate 160 UV–vis spectrophotometer. Polymer solutions (2 mg·mL^–1^ in chloroform) were spin-coated onto glass substrates for optical characterization. The optical bandgap (Eg^opt^) was estimated from the absorption onset wavelength (λ_onset_) according to eq 1,

(E=hc/λ) 1

where h is Planck’s constant (6.626 × 10^–34^ J·s), c is the speed of light (2.998 × 10^8^ m·s^–1^), and λ is the onset wavelength (nm).

Thin-film UV–vis absorption spectra were recorded for all D18 polymers under identical conditions and subsequently normalized to facilitate comparison. The vibronic coupling ratio (A_0_–_0_/A_0_–_1_) was estimated directly from the spectra by identifying the two dominant vibronic features of the lowest-energy absorption band. The A_0_–_0_ transition was assigned to the lowest-energy (longest-wavelength) absorption maximum, while the A_0_–_1_ transition corresponds to the adjacent higher-energy vibronic peak. The absorbance intensities at the maxima of the A_0_–_0_ and A_0_–_1_ transitions were extracted without baseline subtraction or global spectral fitting. The vibronic coupling ratio was then calculated as the ratio between these two absorbance values (A_0_–_0_/A_0_–_1_). This procedure provides a comparative, semiquantitative indicator of intermolecular ordering and aggregation in the solid state and is commonly applied to conjugated polymer thin films.^14,15^

Cyclic voltammetry (CV) measurements were performed using a Metrohm Autolab potentiostat in a 0.1 M tetrabutylammonium hexafluorophosphate (Bu_4_NPF_6_) solution in acetonitrile. A three-electrode configuration was employed, consisting of an ITO-coated glass working electrode, a platinum counter electrode, and an Ag/AgCl reference electrode, at a scan rate of 50 mV·s^–1^. The ferrocene/ferrocenium (Fc/Fc^+^) redox couple was used for internal calibration. The HOMO and LUMO energy levels (eV) were calculated using eqs 2 and 3.

$$\mathrm{HOMO}=-\left({E_{\mathrm{ox}}}^{\mathrm{onset}}+4.4\right)$$ 2

$$\mathrm{LUMO}=\mathrm{HOMO}+{\mathrm{Eg}}^{\mathrm{opt}}3$$ 3

### 2.3 Fabrication of OSC Devices

OSCs were fabricated with using an inverted device architecture: PET/IMI/ZnO/D18:Y6:PC_7__0_BM/PEDOT:PSS/Ag. All solution-processing steps, including ZnO, active layer, and PEDOT:PSS deposition, were performed under ambient atmosphere. In contrast, the Ag top electrode was deposited by thermal evaporation inside a nitrogen-filled MBraun glovebox (O_2_ and H_2_O < 1 ppm), ensuring oxygen- and moisture-free conditions during electrode deposition.

Substrate Preparation. PET/IMI substrates were cleaned sequentially in an ultrasonic bath using Extran detergent solution, acetone, and isopropyl alcohol (15 min each), followed by drying under a nitrogen stream.

#### Film Deposition

All functional layers were deposited in ambient atmosphere by blade coating using a Coatmaster 510 system (Erichsen GmbH, Germany). Film thicknesses were measured using a DektakXT stylus profilometer (Bruker Co., USA). Electron Transport Layer (ETL). The ZnO was deposited from a 2.1 wt % ZnO precursor solution in isopropyl alcohol (InfinityPV ApS, Denmark) using 120 μL of solution, a blade gap of 575 μm, and a coating speed of 5 mm·s^–1^ at a substrate temperature of 45 °C. The films were subsequently annealed at 120 °C for 3 min in air. Active Layer. The active-layer ink consisted of D18:Y6:PC_70_BM (1:1.6:0.2, w/w/w) dissolved in chloroform (11 mg·mL^–1^) with 0.3 vol % 1-chloronaphthalene (CN) as a processing additive. Blade coating was performed using 110 μL of ink, a blade gap of 300 μm, and a coating speed of 60 mm·s^–1^ at 50 °C. The resulting films (≈110 nm thickness) were annealed at 80 °C for 3 min in ambient atmosphere. For solvent-comparison experiments, chloroform was replaced by chlorobenzene or *o*-xylene, while keeping all other processing parameters constant. Hole Transport Layer (HTL). A PEDOT:PSS dispersion (HTL-X, diluted 1:4 v/v in isopropanol) was coated using 120 μL, a 375 μm blade gap, and a speed of 4 mm·s^–1^ at 65 °C, yielding a uniform HTL film. Top Electrode. Ag electrodes were thermally evaporated in a glovebox using a Nexdep 400 (Angstrom Engineering Inc., Canada) under high vacuum (<3 × 10^–6^ mbar) at a deposition rate of 1 Å·s^–1^. The active area of each device was defined as 0.55 cm^2^.

### 2.4 Photovoltaic Characterization

J–V Measurements. Current density–voltage (J–V) characteristics were measured using a Keithley 2420 source meter coupled with a Keithley 2000 multimeter (Keithley Instruments, USA) under simulated AM 1.5G illumination (100 mW·cm^–2^), provided by a solar simulator from Wacom Electric Co., Ltd. (Japan). Light intensity was calibrated with a certified silicon reference cell. Measurements were performed from – 1.0 to +1.0 V with 100 data points per scan.

Optical Characterization. Absorption spectra of active-layer films were obtained using a Shimadzu UV-2600 spectrophotometer (Shimadzu Corporation, Japan) over the 300–1000 nm range. Films were prepared under the same deposition conditions used for device fabrication.

External Quantum Efficiency (EQE). EQE spectra were acquired using a Sciencetech PTS-2-QE IPCE system (Sciencetech Inc., Canada) calibrated with a certified reference diode. Measurements were performed between 280 and 1000 nm using the best-performing device from each fabrication condition.

## 3 Results and Discussion

A series of D18 polymers with distinct and controlled molar masses were synthesized by systematically varying selected Stille polymerization parameters, namely reaction time, palladium catalyst system, and catalyst loading. Rather than establishing comprehensive mechanistic trends, this approach was designed to probe how individual synthetic variables influence chain-growth efficiency and the resulting molar mass distribution under otherwise comparable conditions.

To assess the effect of polymerization duration, reactions were conducted for 12 h (D18–12K) and 24 h (D18–14K) under identical monomer concentrations, solvent, and catalyst system. This comparison enables a direct evaluation of the extent to which prolonged reaction time influences chain extension in this specific Stille coupling system, while acknowledging that reaction time alone does not fully determine molar mass in step-growth polymerizations.

The effect of the palladium catalyst system was investigated by replacing the commonly used Pd_2_(dba)_3_/P­(o-tol)_3_ combination, reported to influence the molecular weight and structural features of conjugated polymers, with Pd­(PPh_3_)_4_, a structurally and electronically distinct Pd(0) precursor.^16^ The resulting polymer (D18–23K) serves as a comparative reference to evaluate relative catalytic efficiency under the present conditions, without implying universal catalyst-dependent structure–property relationships.

To explore the sensitivity of polymer molar mass to catalyst loading, an additional reaction was performed using 25% lower Pd_2_(dba)_3_/P­(o-tol)_3_ concentration relative to the optimized condition. The corresponding polymer (D18–92K) indicates that moderate variations in catalyst loading can significantly influence the achievable molar mass, likely through changes in initiation frequency and chain-termination probability, as previously discussed for Stille polymerizations in conjugated systems.^17,18^ These observations are treated here as empirical correlations rather than definitive mechanistic conclusions.

In addition, a polymer synthesized following a literature-reported high-molar-mass protocol (D18–83K) was prepared and used as an internal benchmark.^3^ This sample provides a reference point for yield, solubility behavior, and molar mass distribution, enabling comparison between optimized, perturbed, and literature-based synthetic conditions within the same experimental framework.

Polymer yields varied substantially across the different synthesis conditions, reflecting differences in solubility, fractionation behavior, and molar mass distribution rather than intrinsic reaction efficiency alone. The isolated yields were 93% for D18–12K (CHCl_3_ fraction), 61% for D18–14K (CHCl_3_ fraction), and 96% for D18–23K (CHCl_3_ fraction). In contrast, the higher molar mass polymers exhibited lower recoveries in the chlorobenzene fraction, with isolated yields of 39% for D18–92K and 65% for D18–83K. These yield variations highlight the strong interplay between molar mass, solvent affinity, and postpolymerization fractionation, which must be considered when comparing isolated masses across different reaction conditions.

A complete summary of polymerization parameters, catalyst systems, isolated fractions, yields, and molar mass values is provided in Table 1, allowing direct and transparent comparison of the synthetic variables explored in this study.

### 3.1 Molar Mass and Dispersity

The results summarized in Table 1 illustrate how variations in selected polymerization parameters, namely reaction time, palladium catalyst system, catalyst loading, and monomer concentration, are associated with measurable differences in the molar mass and dispersity of the synthesized D18 copolymers. Rather than establishing a comprehensive parametric hierarchy, the data highlight the sensitivity of the Stille polymerization of D18 to these variables within the specific experimental space explored in this study.

Extending the reaction time from 12 h (D18–12K) to 24 h (D18–14K) did not result in a significant increase in molar mass under otherwise identical conditions, indicating that, within the experimental window explored here, chain growth is not significantly prolonged by extended heating alone. This observation suggests that the Stille polymerization of D18 may approach kinetic or diffusional limitations at relatively early stages, rather than progressing linearly with reaction time. In contrast, the dispersity increased from 1.83 to 2.18, indicating that prolonged heating favors the development of a broader molar mass distribution. Such behavior is commonly associated with secondary processes, including chain termination, catalyst deactivation, or uneven propagation kinetics, particularly in cross-coupling polymerizations of step-growth character. These effects may be exacerbated at lower monomer concentrations, where the probability of productive monomer–monomer encounters is reduced, leading to less uniform chain growth and broader dispersity.^18,19^

Catalyst identity was also found to influence the achievable molar mass within the conditions investigated. The use of Pd­(PPh_3_)_4_ yielded the polymer D18–23K, exhibiting *M*_n_ and *M*_w_ values approximately 2-fold higher than those obtained for the low-molar-mass batches synthesized using the Pd_2_(dba)_3_/P­(o-tol)_3_ system. This difference is consistent with literature reports showing that mononuclear Pd(0) precursors, such as Pd­(PPh_3_)_4_, provide a more defined coordination environment and higher effective catalytic activity in Stille coupling reactions, thereby facilitating key elementary steps including oxidative addition, transmetalation, and reductive elimination.^20–23^ In line with the well-established behavior of Stille polycondensations, both monomer concentration and catalyst efficiency play critical roles in determining the overall degree of polymerization.^24^ The slightly lower dispersity of D18–23K (Đ = 1.92) relative to D18–14K (Đ = 2.18) suggests a more uniform chain-growth process under these specific conditions. However, given the limited number of data points, these observations are treated as empirical correlations rather than evidence of a general catalyst-dependent mechanistic effect. Notably, even with improved catalytic efficiency, the molar masses achieved using Pd­(PPh_3_)_4_ remained below those of the highest molar mass samples, indicating that catalyst identity alone is insufficient to maximize chain length.

The highest molar mass polymer in this series, D18–92K, was obtained when the loading of the Pd_2_(dba)_3_/P­(o-tol)_3_ catalyst system was reduced by 25%. Although this result may appear counterintuitive, similar behavior has been reported for cross-coupling polymerizations, in which a lower density of active catalytic sites can suppress premature initiation and termination events, thereby allowing individual polymer chains to propagate for longer periods before deactivation.^22,25^ In the present case, the increased *M*_w_ is accompanied by a lower isolated yield and a relatively broad dispersity (Đ = 2.18), highlighting an intrinsic trade-off between maximizing molar mass and maintaining efficient polymer recovery. This broader molar mass distribution also suggests the coexistence of very long chains with shorter fractions, which can influence film uniformity and processing behavior.

By contrast, the literature-standard synthesis conducted at higher monomer concentration yielded the benchmark D18–83K sample (*M*_n_ = 42.65 kg·mol^–1^; *M*_w_ = 83.66 kg·mol^–1^) with a narrower dispersity (Đ = 1.98) and higher isolated yield. Within the limited parameter space explored in this study, this comparison indicates that monomer concentration plays an important role in governing chain growth and overall polymerization efficiency, in agreement with prior reports on Stille-coupled conjugated polymers.^23^ However, this observation should be interpreted cautiously, as the present results also demonstrate that catalyst loading, dispersity, and fractionation behavior collectively influence the final molar mass characteristics. Accordingly, no single synthetic parameter can be regarded as universally dominant, and optimal D18 synthesis requires balancing molar mass maximization, dispersity control, and material recovery.

Collectively, these results demonstrate that reaction time, catalyst identity, catalyst loading, and monomer concentration act as complementary synthetic parameters for systematically tuning the molar mass and dispersity of D18 within the conditions investigated. These parameters govern solubility, aggregation, and film formation, thereby critically influencing the morphology and photovoltaic performance of donor–acceptor polymer blends, as discussed in the following sections.

### 3.2 Optoelectronic Properties

The optical and electrochemical characteristics of the synthesized D18 polymers are summarized in Table 2. Normalized UV–vis absorption spectra, shown in Figure 1, were acquired from thin films prepared under identical blade-coating conditions, allowing a direct comparison of spectral features and line shapes. Normalized spectra are presented to facilitate qualitative comparison of vibronic structure and aggregation signatures, while non-normalized absorption spectra, obtained from films prepared under the same deposition conditions and comparable thickness range, are provided in the Supporting Information (Figure S1) to support discussions related to absorption intensity.

**Table 2:** Optical and Electrochemical Properties of D18 Copolymers

	cyclic voltammetry		UV–vis		
**polymer**	^a^ **HOMO (eV)**	^b^ **LUMO (eV)**	**λ**_**max**_ **(nm)**	**λ**_**onset**_ **(nm)**	^c^ **E**_**g**_^**opt**^ **(eV)**
D18–12K	–5.64	–3.71	546/576	642	1.93
D18–14K	–5.63	–3.70	548/574	641	1.93
D18–23K	–5.62	–3.65	526/570	629	1.97
D18–92K	–5.68	–3.76	554/586	642	1.93
D18–83K	–5.44	–3.48	546/582	644	1.96

a Estimated from cyclic voltammetry (CV) measurements.

b Calculated according to LUMO = HOMO + Eg^opt^.

c Optical bandgap (Eg^opt^) calculated from the absorption onset wavelength using the empirical relation Eg^opt^ (eV) = 1240/λ_onset_ (nm).

**Figure 1:**
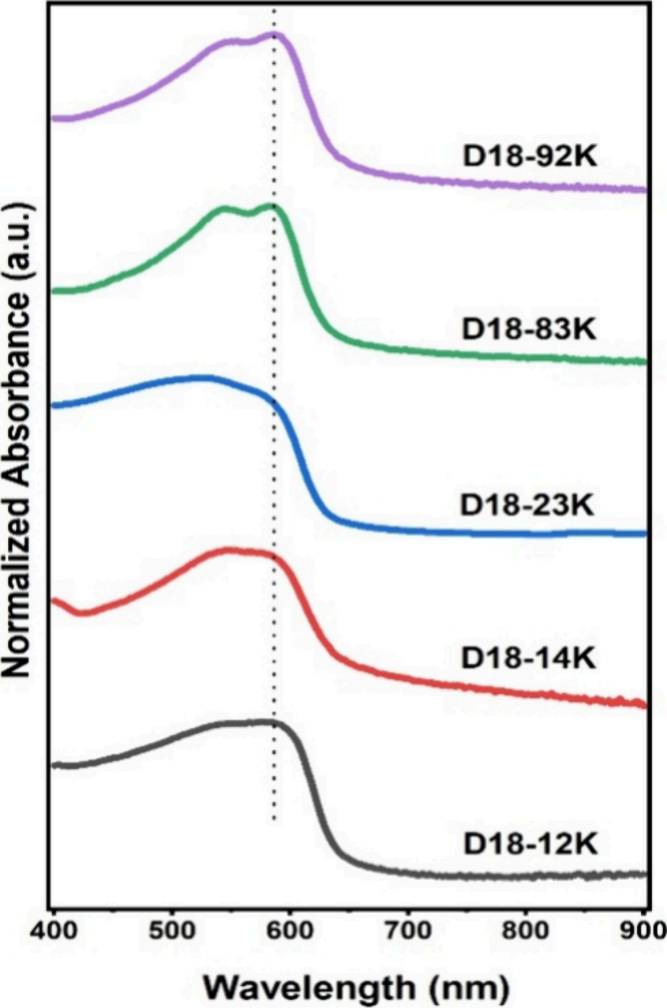
Normalized UV–vis absorption spectra of D18 samples with different molar masses.

The electrochemical measurements reveal that the frontier orbital energy levels remain relatively stable across the series, with HOMO values ranging from – 5.44 to – 5.68 eV, which lies within the optimal energy window for achieving high open-circuit voltages (*V*_oc_) in donor–acceptor nonfullerene OSCs.^3,19^ The limited variation in HOMO and LUMO levels suggests that changes in polymerization conditions primarily affect molar mass and solid-state organization, rather than significantly perturbing the intrinsic electronic structure of the D18 conjugated backbone.

Within the polymer series, D18–83K, synthesized under literature-standard conditions and exhibiting one of the highest molar masses, displayed slightly higher (less negative) HOMO (−5.44 eV) and LUMO (−3.48 eV) energy levels. This modest shift may be associated with differences in solid-state organization, as higher molar mass samples typically exhibit enhanced chain packing and aggregation, which can favor greater effective π-electron delocalization in the film state.^26–28^ However, given the limited magnitude of the energy-level variation, these effects are interpreted as subtle and indirect rather than as evidence of a distinct electronic structure modification.

Conversely, the lower molar mass samples (D18–12K and D18–14K) exhibited marginally deeper HOMO levels, which is consistent with less ordered films and a higher degree of electronic localization, as commonly reported for conjugated polymers with reduced chain length.

Most polymers exhibited an optical bandgap (Eg^opt^) of approximately 1.93 eV, which is slightly smaller than the value typically reported for commercial D18 (∼1.98 eV). This observation indicates that, although polymerization conditions significantly affect molar mass and solid-state organization, they exert only a limited influence on the intrinsic electronic structure of the D18 chromophore.

The most noticeable deviation was observed for D18–23K, synthesized using Pd­(PPh_3_)_4_, which exhibited a slightly larger optical bandgap (Eg^opt^ = 1.97 eV). Rather than attributing this shift to a specific catalyst-induced backbone planarization effect, the increase is more conservatively ascribed to the combined influence of lower molar mass (*M*_w_ = 23.71 kg·mol^–1^) and reduced interchain aggregation, both of which are known to limit effective conjugation length and enhance spectral blue shifts in thin films. ^30^

The UV–vis absorption spectra provide direct evidence of molar-mass-dependent ordering in the solid state. High-molar-mass polymers, such as D18–92K and D18–83K, exhibit well-resolved vibronic structures with dual absorption maxima at 554/586 nm and 546/582 nm, respectively. These features are characteristic of donor–acceptor (D–A) alternating copolymers with enhanced interchain π–π stacking and aggregation, leading to enhanced delocalization and absorption in the lower-energy region of the spectrum.^29^ The slight red shift observed for D18–92K further suggests extended effective conjugation enabled by longer polymer chains and improved intermolecular interactions.

In contrast, low-molar-mass polymers display broader and less structured absorption profiles. D18–12K (λ_max_ = 576 nm) and D18–14K (λ_max_ = 548 nm) show blue-shifted, featureless spectra indicative of disordered packing, reduced exciton delocalization, and shorter effective conjugation lengths. Such spectral characteristics are commonly associated with limited chain overlap and conformational disorder, conditions that are detrimental to charge transport and exciton diffusion.^19^ These optical signatures are fully consistent with the inferior photovoltaic performance observed for the low-molar-mass batches.

A distinct spectral behavior is observed for D18–23K, synthesized using Pd­(PPh_3_)_4_ instead of the Pd_2_(dba)_3_/P­(o-tol)_3_ catalytic system. This polymer exhibits a broad absorption band with only a weak vibronic shoulder, markedly different from the structured spectra of the higher-*M*_w_ samples. Rather than attributing this behavior to a definitive catalyst-induced backbone planarization effect, the observed spectral broadening are more conservatively ascribed to the combined influence of moderate molar mass (*M*_w_ = 23.71 kg·mol^–1^) and reduced interchain aggregation. Both factors are known to limit long-range ordering and suppress vibronic resolution in conjugated polymer films.

The vibronic coupling ratio (A_0_–_0_/A_0_–_1_) serves as an indirect indicator of intermolecular ordering in conjugated polymer films, as an enhanced A_0_–_0_ contribution reflects stronger π–π stacking and increased electronic coherence between polymer chains. To provide a more quantitative assessment of aggregation effects, the vibronic intensity ratio A_0_–_0_/A_0_–_1_ was extracted from the normalized absorption spectra (Table S1). The ratios exhibit only moderate variations across the D18 series, indicating that changes in molar mass do not induce a strong monotonic evolution of vibronic coupling. While the low-*M*_w_ samples (D18–12K and D18–14K) show ratios close to unity, a reduced value is observed for the intermediate-*M*_w_ polymer (D18–23K), suggesting weaker vibronic coupling and less pronounced aggregation. In contrast, the highest-*M*_w_ samples (D18–83K and D18–92K) display slightly higher A_0_–_0_/A_0_–_1_ ratios, pointing to a modest enhancement of backbone planarization and interchain ordering. Overall, these results indicate that molar mass primarily influences aggregation in a subtle manner, consistent with the observed morphological trends in blade-coated films rather than inducing drastic changes in electronic structure.

Despite these differences in aggregation signatures and spectral line shapes, the optical bandgap remains relatively constant across the polymer series. This indicates that the intrinsic electronic structure of the D18 repeat unit is largely preserved and that variations in optical response and device performance are predominantly governed by differences in solid-state organization and chain packing rather than by fundamental changes in frontier orbital energies.

Overall, these results indicate that molar mass plays a key role in modulating the solid-state organization and photophysical response of D18 polymers. Rather than inducing a strong linear increase in aggregation strength, higher molar masses are associated with subtle improvements in vibronic ordering and film organization, which are sufficient to impact charge transport and photovoltaic performance. This highlights the importance of molar mass control as a processing–structure parameter in defining the device-relevant properties of donor polymers in bulk-heterojunction organic solar cells.

### 3.3 Photovoltaic Performance

#### 3.3.1 J–V Characteristics

The photovoltaic performance of the D18 copolymers with different molar masses was evaluated using an inverted BHJ device architecture PET/IMI/ZnO/D18:Y6:PC_70_BM/PEDOT:PSS/Ag, fabricated by blade coating with a large active area of 0.55 cm^2^. A commercial D18 sample (D18C-92K) was used as a reference. All solution-processed layers were deposited under ambient atmosphere; only top electrode Ag evaporation was performed under inert conditions.The key performance parameters, including *V*_oc_, *J*_sc_, FF, PCE, series resistance (R_s_), and shunt resistance (R_sh_), are summarized in Table 3. A clear dependence of device efficiency on polymer molar mass was observed. As Mw increased, devices exhibited systematic improvements in *J*_sc_ and FF, leading to higher overall PCE values (Figure 2). The highest-*M*_w_ polymers, D18–92K and D18–83K, delivered PCEs of 7.77% and 7.99%, respectively, approaching the performance of commercial D18C-92K (8.94%). These results indicate that increasing chain length is associated with improved BHJ film quality and electrical uniformity, which are consistent with more efficient charge generation and extraction, rather than directly evidencing intrinsic changes in charge-transport properties.^30^

**Table 3:** Device Data of OSCs Based on D18 Copolymers^a^

photovoltaic parameters of devices						
polymer donor	*V*_oc_ (V)	*J*_sc_ (mA cm^–2^)	FF (%)	PCE (%)^b^	R_s_ (Ohm)	R_sh_ (Ohm)
D18–12K	0.732 (0.549 ± 0.230)	7.40 (6.30 ± 1.37)	40.5 (33.58 ± 4.92)	1.96 (1.31 ± 0.63)	62	382
D18–14K	0.794 (0.785 ± 0.014)	5.25 (4.80 ± 0.26)	41.0 (37.05 ± 1.98)	1.56 (1.39 ± 0.09)	101	414
D18–23K	0.780 (0.764 ± 0.010)	6.31 (5.98 ± 0.22)	44.7 (43.08 ± 0.94)	2.15 (1.97 ± 0.10)	73	534
D18–92K	0.813 (0.809 ± 0.002)	14.30 (13.92 ± 0.21)	67.2 (65.07 ± 1.77)	7.77 (7.33 ± 0.22)	17	2157
D18–83K	0.815 (0.809 ± 0.003)	14.44 (13.55 ± 0.37)	69.6 (67.42 ± 2.30)	7.99 (7.39 ± 0.30)	16	2239
^b^D18C-92K	0.823 (0.818 ± 0.004)	16.20 (14.37 ± 1.22)	69.40 (66.42 ± 1.61)	8.94 (7.81 ± 0.72)	18	2405

a Commercial D18 reference sample (*M*_n_ ≈ 41 kg·mol^–1^, *M*_w_ ≈ 92 kg·mol^–1^).

b Device configuration: Donor: D18; nonfullerene acceptor (NFA): Y6; fullerene acceptor (FA): PC_70_BM. Blend ratio (donor:NFA:FA, w/w/w): 1:1.6:0.2, with 0.3 vol % 1-chloronaphthalene (CN) as a processing additive in chloroform. Thermal annealing: 80 °C for 3 min. Active-layer thickness: 80–110 nm. The reported values correspond to the best-performing device for each system; values in parentheses represent the average ± standard deviation, calculated from eight independent devices.

**Figure 2:**
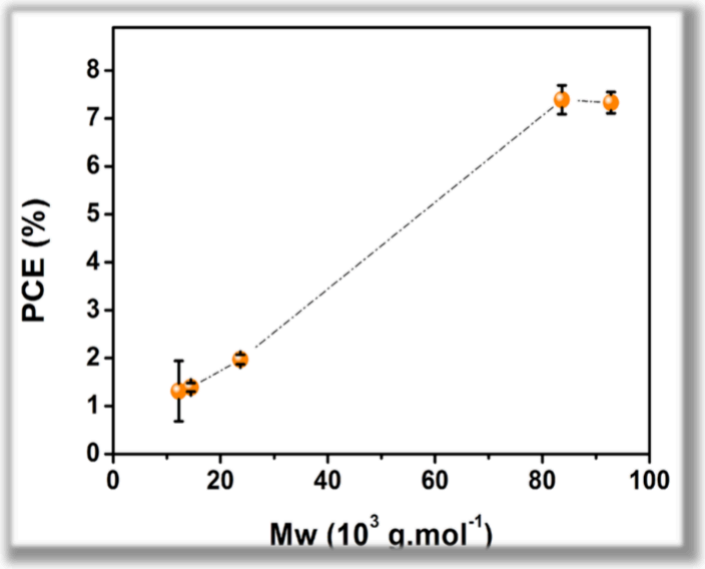
Influence of molar mass (*M*_w_) on energy efficiency (PCE).

The origin of this behavior can be qualitatively rationalized by the structural characteristics of high-*M*_w_ copolymers. Longer polymer chains are commonly associated with enhanced intermolecular π–π stacking, improved solid-state ordering, and more continuous percolation pathways for charge transport.

Such traits are consistent with increased exciton dissociation efficiency, facilitated long-range hole transport, and reduced trap-assisted recombination, in line with the large increases in *J*_sc_ and FF observed for D18–83K and D18–92K. In contrast, low-*M*_w_ samples (D18–12K and D18–14K) tend to exhibit poor BHJ connectivity and limited ordering, resulting in reduced *J*_sc_ (≤7.4 mA·cm^–2^), FF (<41%), and PCE (<2%).

The extremely low shunt resistance of the low-*M*_w_ devices (R_sh_ < 400 Ω·cm^2^) indicates parasitic leakage currents, which are likely associated with morphological defects such as pinholes, nonuniform film coverage, or rough interfaces. Such features are consistent with the disordered optical spectra and low absorption intensities previously observed for these samples.^31^ Conversely, the high-*M*_w_ polymers displayed Rsh values exceeding 2,000 Ω·cm^2^, directly contributing to elevated FF values (69.6% for D18–83K and 67.2% for D18–92K). These high R_sh_ values indicate suppressed leakage currents and improved film integrity, reinforcing the importance of molar mass in determining active-layer morphology and electrical quality. The series resistance varied only modestly across the devices but was generally lower in the highest-performing samples, which is consistent with more efficient charge extraction.

The J–V curves (Figure 3a) clearly illustrate the impact of polymer molar mass on device behavior. Devices based on D18–12K and D18–14K exhibit flattened J–V characteristics, shallow slopes near the maximum power point, and pronounced curvature in the fourth quadrant, signatures commonly associated with inefficient charge transport and high recombination losses. D18–23K, synthesized using Pd­(PPh_3_)_4_, shows moderate improvements but still lacks the diode-like profile of high-performing devices, suggesting limitations imposed by its lower chain length and reduced solid-state ordering. In contrast, devices incorporating D18–83K, D18–92K, and commercial D18C-92K show steep J–V rises, high short-circuit current densities, and saturating behavior under forward bias, indicative of efficient charge collection and minimal resistive losses.

**Figure 3:**
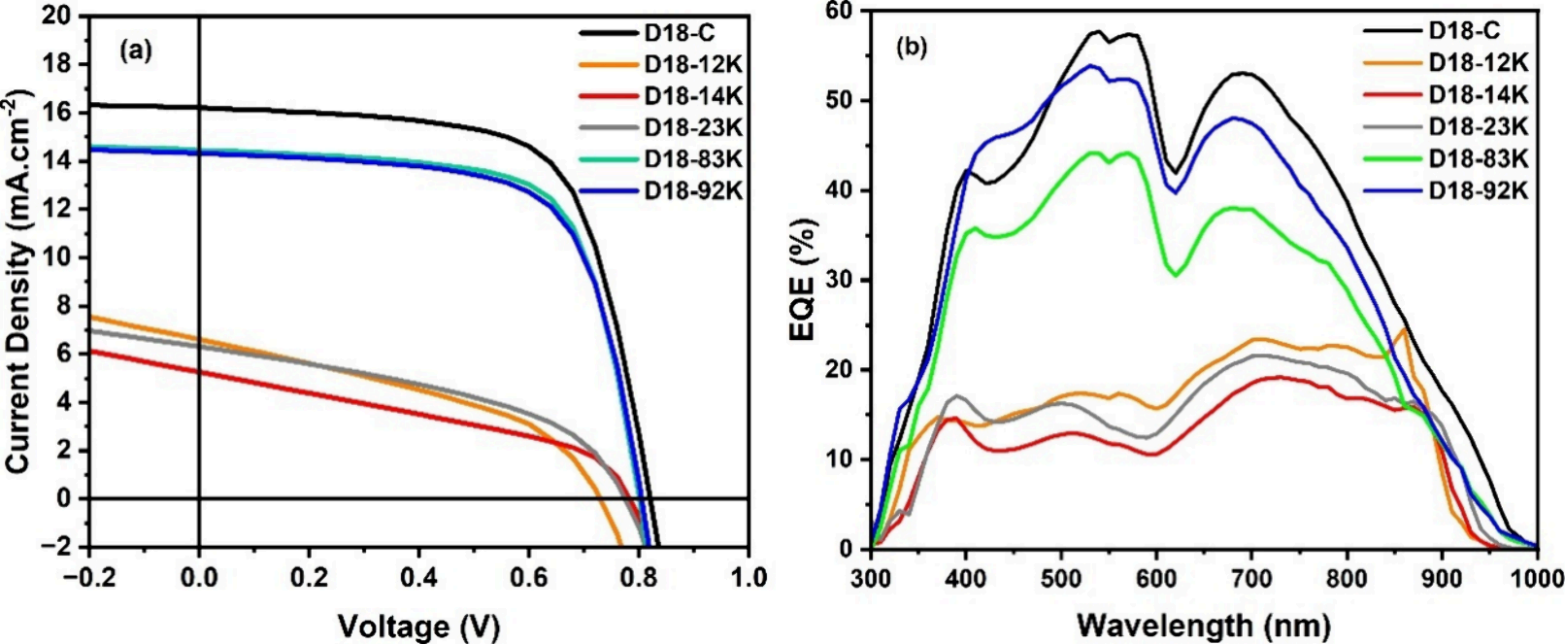
(a) Voltage–current curves; (b) external quantum efficiency (EQE) spectrum for D18-based OSC prepared in chloroform.

External quantum efficiency (EQE) spectra (Figure 3b) further support the trends observed in J–V measurements. Devices fabricated with high-*M*_w_ polymers exhibit substantially higher EQE values across the full 300–950 nm range, including the near-infrared region where D18 absorption is dominant. The broad and intense EQE responses are consistent with enhanced light harvesting, efficient exciton diffusion, and more favorable charge-generation dynamics within the BHJ. Consistent with these findings, the high-*M*_w_ polymers showed superior *J*_sc_ values, supporting the conclusion that increased chain length and improved intermolecular organization play a critical role in maximizing photocurrent generation.

In contrast, low-*M*_w_ devices exhibit weaker, less structured EQE spectra, particularly beyond 700 nm. These diminished responses are consistent with limited conjugation length, increased energetic disorder, and less effective percolation pathways associated with short donor chains. Such microstructural deficiencies are commonly associated with increased recombination losses and reduced charge-collection efficiency, thereby contributing to the low performance of these devices.

Overall, the combined J–V and EQE analyses demonstrate that molar mass is a primary determinant of D18-based device performance. Increasing *M*_w_ is associated with enhanced molecular ordering, improved phase continuity, and more effective charge-transport pathways, leading to higher *J*_sc_, higher FF, and ultimately higher PCE. These results underscore that *M*_n_, *M*_w_, and Đ, controlled synthetically during polymerization, play a decisive role in defining device-relevant morphology and electrical performance and therefore must be carefully optimized to enable high-performance, solution-processed organic photovoltaic devices.

#### 3.3.2 Solvent Selection for the Preparation of OSC Devices

Selecting an appropriate solvent is a key requirement in the fabrication of organic solar devices, as the miscibility, solubility, and drying behavior of the donor and acceptor components strongly influence the final active-layer morphology. In addition to enabling desirable nanoscale phase separation, the solvent must comply with increasingly strict environmental and industrial guidelines, creating demand for low-toxicity and nonhalogenated processing alternatives suitable for large-scale manufacturing.

In this study, three solvents with distinct physicochemical profiles, chlorobenzene (CB), chloroform (CF), and *ortho*-xylene (*o*-xylene), were evaluated for preparing active layers based on both a low-molar-mass polymer (D18–12K) and a high-molar-mass polymer (commercial D18C-92K). These solvents differ in polarity and boiling point, parameters that are known to strongly influence solubility, aggregation kinetics, film formation, and ultimately device performance.

The photovoltaic parameters obtained with each solvent system are summarized in Table 4. A clear dependence on both polymer molar mass and solvent choice was observed. For the high-molar-mass donor (D18C-92K), devices processed using halogenated solvents (CF and CB) delivered substantially higher *J*_sc_ and improved EQE responses than those processed in *o*-xylene. This trend is consistent with more favorable blend morphology and charge-transport networks when the donor is sufficiently dissolved, and the drying kinetics allow molecular organization.

**Table 4:** Device Data of OSCs Based on D18 Copolymers in Chlorobenzene and *o*-Xylene^a^

photovoltaic parameters of devices						
polymer donor	*V*_oc_ (V)	*J*_sc_ (mA cm^–2^)	FF (%)	PCE (%)^a^	R_s_ (Ohm)	R_sh_ (Ohm)
**Chlorobenzene**						
D18–12K	0.768 (0.758 ± 0.010)	11.4 (10.83 ± 0.36)	46.0 (43.06 ± 2.38)	3.92 (3.55 ± 0.33)	37	371
D18C-92K	0.788 (0.780 ± 0.010)	16.9 (8.93 ± 0.70)	67 (58.49 ± 7.01)	8.94 (7.81 ± 1.19)	17	1706
***o*-Xylene**						
D18–12K	0.767 (0.727 ± 0.034)	11.0 (10.21 ± 0.38)	43.6 (39.29 ± 2.74)	3.40 (2.92 ± 0.32)	49	366
D18C-92K	0.680 (0.625 ± 0.064)	10.1 (8.93 ± 0.60)	48.9 (42.03 ± 5.61)	3.11 (2.39 ± 0.59)	38	482

a Device configuration: Donor: D18; nonfullerene acceptor (NFA): Y6; fullerene acceptor (FA): PCBM. Blend ratio (donor:NFA:FA, w/w/w): 1:1.6:0.2, with 0.3 vol % 1-chloronaphthalene (CN) as a processing additive in the respective solvent. Thermal annealing: 80 °C for 3 min. Active-layer thickness: 80–110 nm. The reported values correspond to the best-performing device for each system; values in parentheses represent the average ± standard deviation obtained from eight independent devices.

To rationalize the solvent- and molar-mass–dependent morphology observed in Figure 4, it is useful to consider the thermodynamics of polymer solutions as described by the Flory–Huggins theory. In this framework, film formation and aggregation behavior arise from a balance between the entropic contribution associated with polymer chain configuration and the enthalpic interactions between polymer and solvent species. Recent studies have shown that, when evaluated alongside experimental observations, Flory–Huggins interaction parameters provide valuable qualitative insight into polymer solubility, aggregation, and film-forming behavior in conjugated polymer systems.^32,33^

**Figure 4:**
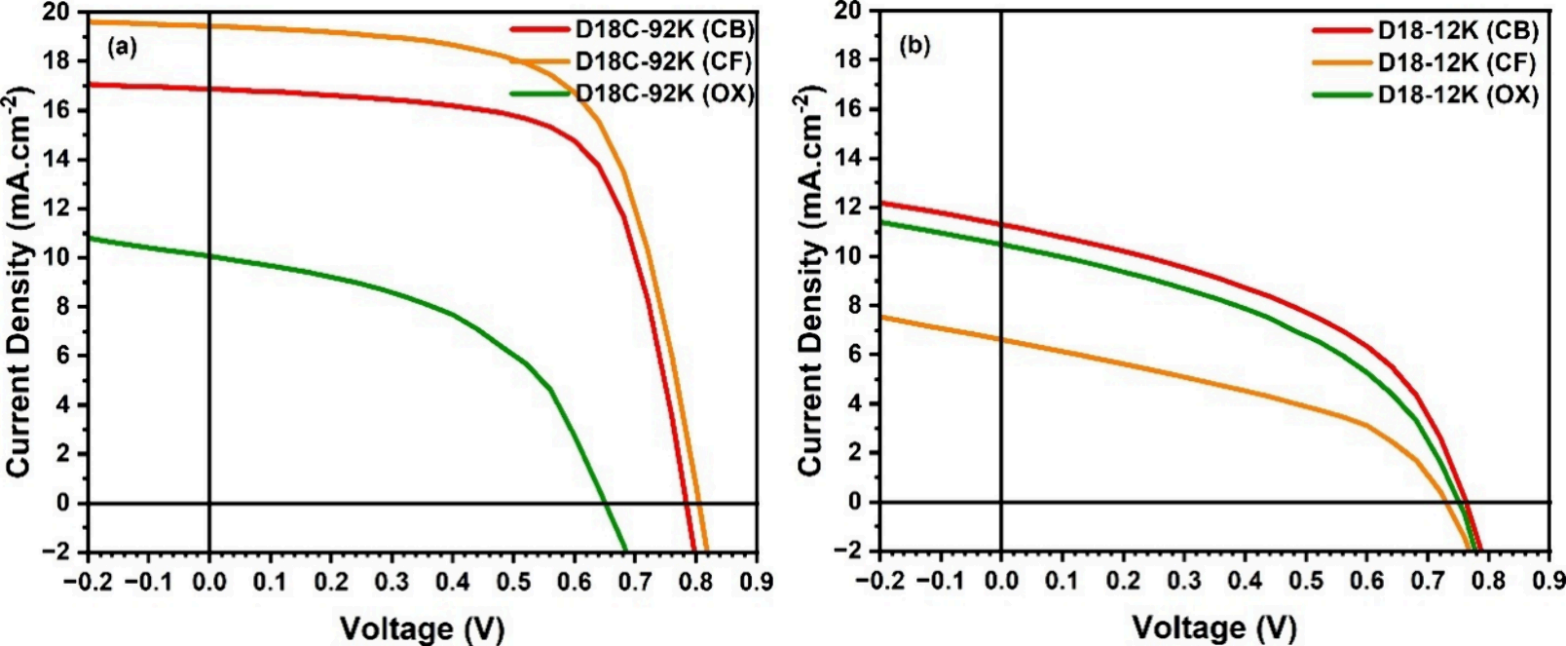
Voltage–current curves of devices prepared in different solvents, chloroform (CF), chlorobenzene (CB), and *o*-xylene (ox): (a) commercial D18C-92K; (b) D18–12K.

In particular, Lu et al.^32^ reported that polymers exhibiting limited solubility in a given solvent can develop distinct aggregation and packing motifs during film formation, depending strongly on molar mass and chain regularity. In the present case, D18 displays reduced solubility in *o*-xylene, and this effect is mitigated for lower molar mass samples, which exhibit shorter effective conjugation lengths and reduced crystallization propensity. As a result, excessive preaggregation is suppressed, promoting more homogeneous donor–acceptor intermixing.

Furthermore, Qiu and co-workers^33^ demonstrated that solvent evaporation kinetics play a critical role in defining aggregation pathways during solution casting. For marginal solvents such as *o*-xylene, lower molar mass polymers experience slower and more controlled aggregation, leading to fibrillar morphologies that favor efficient bulk heterojunction interfaces. This framework is fully consistent with the morphological trends observed here, where low-*M*_w_ D18 processed from *o*-xylene exhibits improved blend homogeneity relative to its high-*M*_w_ counterpart.

In contrast, for the low-molar-mass polymer (D18–12K), the solvent effect is reversed. Devices fabricated using *o*-xylene exhibit performance comparable to, or even surpassing, those processed from halogenated solvents. EQE spectra (Figure 5) show a more balanced response across the visible–near-infrared region, which is consistent with a more homogeneous morphology. Because low-*M*_w_ chains typically display higher solubility and reduced aggregation propensity in *o*-xylene, the resulting films tend to contain smaller domains and improved donor–acceptor intermixing. This morphology is generally favorable for efficient charge separation and transport, thereby contributing to enhanced device performance.

**Figure 5:**
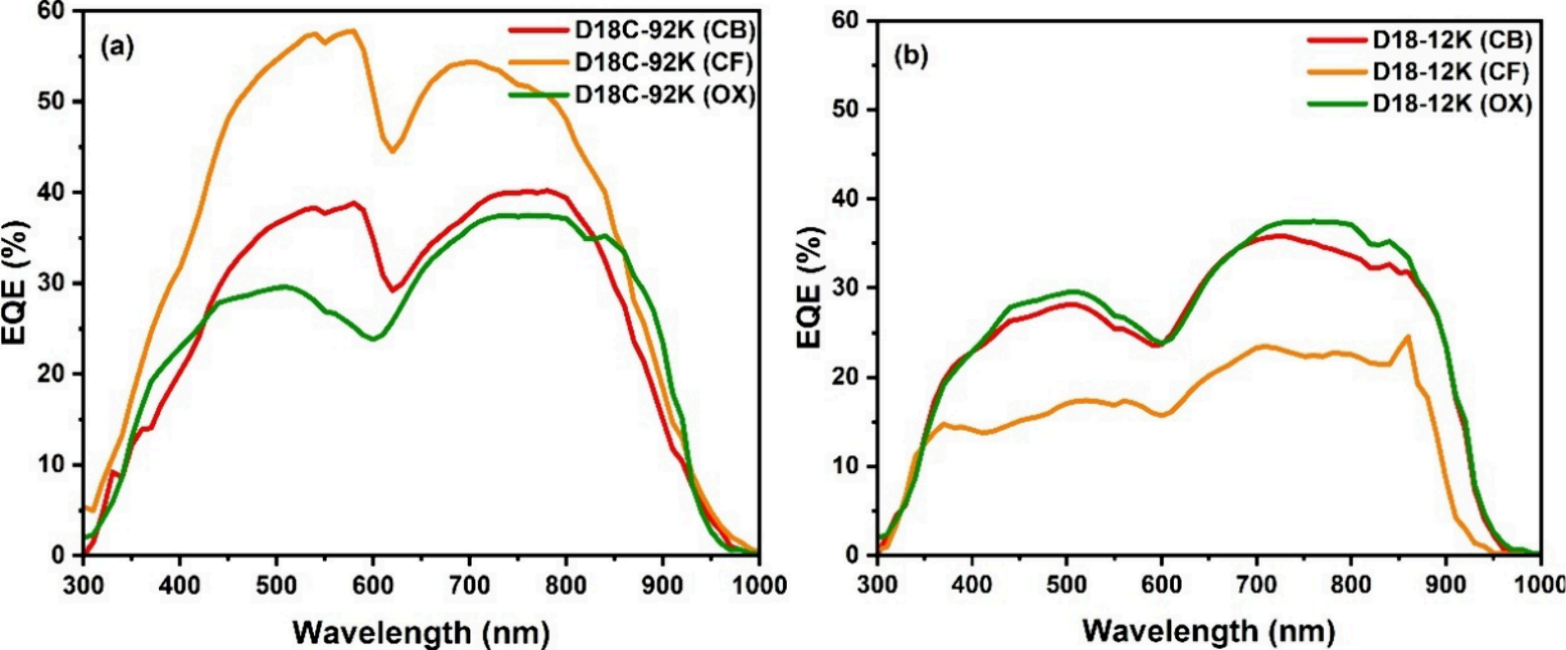
External quantum efficiency (EQE) spectrum for D18-based OSC prepared in different solvents, chloroform (CF), chlorobenzene (CB), and *o*-xylene (OX): (a) commercial D18C-92K; (b) D18–12K.

Together, these results highlight that solvent selection must be tailored to polymer molar mass. While high-*M*_w_ donors often require halogenated solvents to avoid uncontrolled aggregation and ensure optimal packing, low-*M*_w_ donors can be effectively processed using greener, nonhalogenated solvents such as *o*-xylene. This molecular-weight–dependent solvent compatibility provides a practical framework for designing scalable and environmentally friendly fabrication protocols for next-generation organic solar cells.

In *o*-xylene, the limited solubility and higher viscosity of high-*M*_n_ polymer solutions are associated with excessive preaggregation and the formation of large, heterogeneous domains, as observed in the optical microscopy results presented in Table 5. Such coarse morphology is commonly linked to limited exciton dissociation and leads to inefficient charge extraction, in line with previously reported solvent-dependent morphology trends for high-*M*_w_ donor–acceptor systems.^29^

**Table 5:**
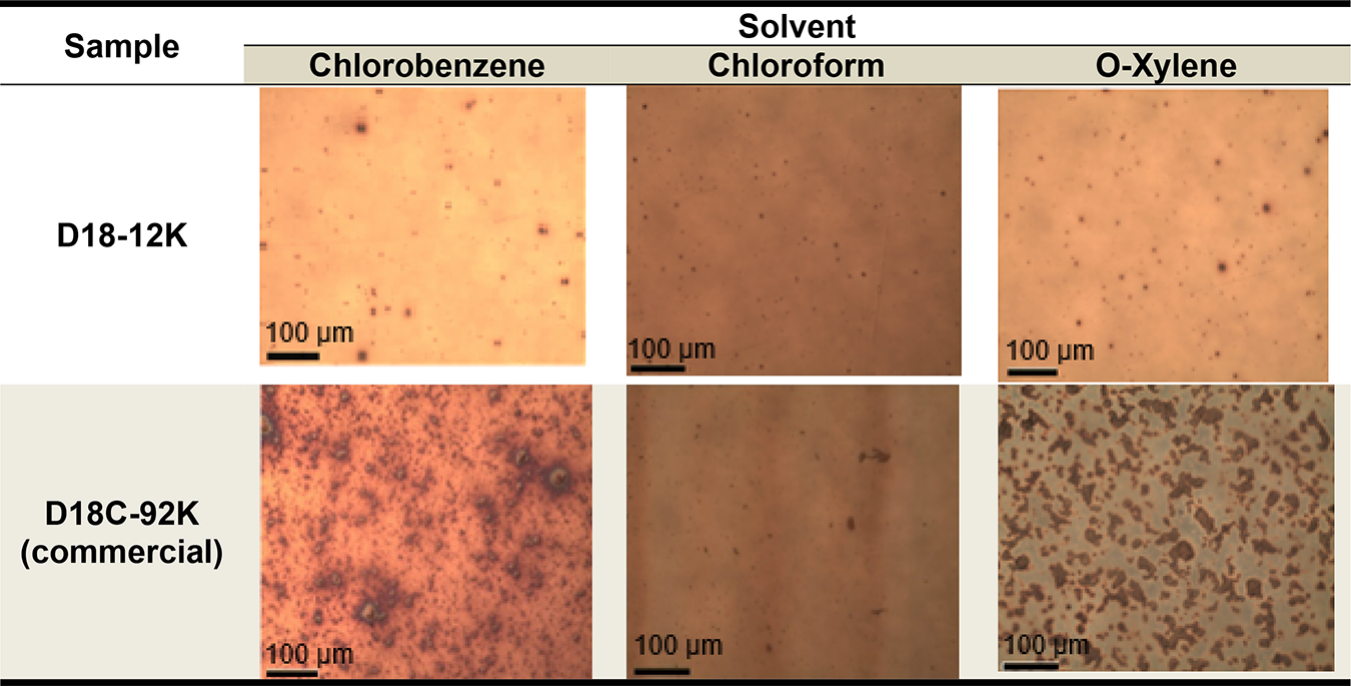
Optical Microscopy Images of the Active Layer of the Devices (10× Magnification)

#### 3.3.3 AFM Morphology Analysis

Figure 6 shows AFM topography images (5 × 5 μm^2^) of the active layers processed with (a) the low-molecular-weight D18–12K (*M*_w_ = 12.22 kg·mol^–1^) and (b) the high-molecular-weight D18–92K (*M*_w_ = 92.79 kg·mol^–1^). A pronounced contrast in nanoscale morphology and surface roughness is observed between the two films, highlighting the strong influence of polymer chain length on bulk heterojunction (BHJ) organization.

**Figure 6:**
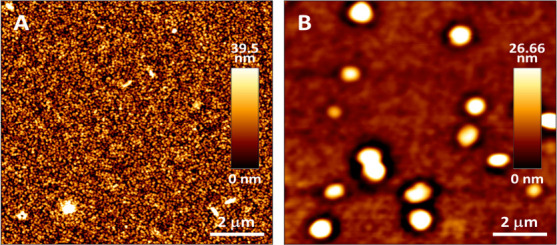
AFM topography images (5 × 5 μm^2^) of the active layer composed of D18:Y6:PC_7__0_BM processed (chloroform) with (A) low-molar-mass polymer D18–12K (*M*_w_ = 12.22 kg·mol^–1^); (B) high-molar-mass polymer D18–92K (*M*_w_ = 92.79 kg·mol^–1^).

The D18–12K-based film (Figure 6a) exhibits a relatively fine-grained and homogeneous surface, marked by low phase contrast and small, poorly defined domains. This morphology is consistent with limited phase separation and reduced structural ordering, features commonly associated with short polymer chains that are less effective at promoting long-range ordering or robust donor–acceptor segregation. Such disordered nanostructures are typically linked to restricted exciton dissociation and hindered charge percolation, in agreement with the reduced EQE response, lower *J*_sc_, and overall poor photovoltaic performance observed for devices incorporating D18–12K.

In contrast, the high-molecular-weight D18–92K film (Figure 6b) presents a more heterogeneous topography, with larger domains and stronger height and phase contrast. These morphological features suggest enhanced π–π stacking and more efficient self-organization, which are commonly associated with increased chain entanglement and stronger intermolecular interactions characteristic of high-*M*_w_ conjugated polymers. Although some degree of domain coarsening is present, the domain sizes remain within a range typically reported as favorable for BHJ solar cells^19^ allowing a balance between donor–acceptor demixing with sufficient interfacial area for efficient exciton dissociation.

Despite the more prominent vertical features in the D18–92K film, its overall RMS roughness is lower. The height scale bars reveal maximum height variations of ∼ 39.5 nm for D18–12K and ∼ 26.6 nm for D18–92K. Complementary profilometry confirmed this trend: the low-*M*_w_ and high-*M*_w_ films displayed average roughness values of 5.32 ± 2.98 nm and 3.07 ± 1.07 nm, respectively. The smoother and more compact morphology of the high-*M*_w_ film is consistent with improved vertical charge-transport pathways, higher shunt resistance, and enhanced fill factor, in agreement with the photovoltaic trends discussed earlier.

Overall, AFM analyses support the conclusion that increasing the polymer molar mass is associated with more favorable nanostructural organization within the BHJ, characterized by improved donor–acceptor phase separation, enhanced molecular packing, and the formation of more continuous and efficient percolation pathways. These morphological refinements are closely correlated with the superior optoelectronic properties and device performance observed for high-Mw D18 donors, highlighting the central role of chain length–driven self-assembly in governing OSC efficiency. Consistently, higher-Mw D18 samples exhibit systematically increased A_0_–_0_/A_0_–_1_ ratios, in agreement with their more structured UV–vis absorption features, and the enhanced charge transport and collection reflected in the J–V and EQE characteristics.

## 4 Conclusions

This work demonstrates that the polymerization conditions of D18 are closely associated with pronounced variations in molar mass and, consequently, with the photovoltaic performance of organic solar cells. By systematically tuning the catalyst system, reaction time, and monomer concentration, D18 polymers with *M*_w_ values ranging from 12 to 92 kg·mol^–1^ were obtained, enabling a clear assessment of the relationship between polymer chain length, morphological organization, and optoelectronic behavior.

Low-molecular-mass polymers exhibited comparable optical bandgaps and frontier energy levels but yielded low-efficiency devices (≈2%). This limited performance is consistent with restricted chain connectivity and suboptimal film organization, as evidenced by UV–vis absorption, EQE response, and AFM morphology analyses. In contrast, high-molecular-mass polymers achieved markedly improved device efficiencies (≈7.7–7.9%), approaching the performance of commercial D18, highlighting the critical role of chain length in enabling favorable solid-state organization.

The solvent-selection study further emphasizes the interplay between polymer molar mass and processing conditions. For high-*M*_w_ polymers, halogenated solvents (CF and CB) were associated with improved solubility, controlled aggregation, and efficient charge transport, resulting in the highest device efficiencies. Conversely, the greener nonhalogenated solvent *o*-xylene proved more suitable for low-*M*_w_ polymers, yielding more homogeneous morphologies and enhanced device response. These results demonstrate that solvent choice must be tailored to polymer chain length to achieve optimal active-layer structures.

AFM analyses are fully consistent with these trends, revealing that high-*M*_w_ polymers form smoother, more compact, and better-organized films, whereas low-*M*_w_ materials exhibit rougher surfaces and less favorable phase separation. Collectively, these findings confirm that molar mass plays a central role in shaping active-layer morphology and is closely linked to device efficiency in D18-based systems.

Overall, this study highlights that the combined optimization of polymer molar mass and processing conditions is essential for enhancing the performance of D18-based donor materials. The insights provided here offer practical guidance for scalable, blade-coated device fabrication and contribute to a deeper understanding of structure–processing–property relationships in high-performance organic photovoltaic polymers.

## Supplementary Material



## Notes

Additional UV–vis spectra of thin films (Figure S1), vibronic coupling analysis and A_0_–_0_/A_0_–_1_ ratio evaluation (Table S1), and supplementary experimental details related to polymer characterization and photovoltaic device analysis (PDF)
