# Supplementary material for: Tuning Molar Mass of the D18 Polymer via Stille Polymerization: Impact on Morphology and Large-Area Blade-Coated Organic Solar Cells
Source: ACS Omega. 2026 Mar 2;11(10):16489–500. doi: 10.1021/acsomega.5c12462 (PMC13000590; doi:10.1021/acsomega.5c12462)
Supplement: Supplementary file 1 [file ao5c12462_si_001.pdf]

Tuning Molar Mass of the D18 Polymer via Stille Polymerization: Impact on Morphology and Large-Area Blade-Coated Organic Solar Cells

[1]Renata S. Cardoso, [1]Igor T. Soares, [1]João A. F. L. Batalha, [1]Isabela C. Mota, [1]Lucas G. P. Tienne, [1]Tamires Y. G. Alves, [1]Letícia A. Marcate, [2]Juliana L. S. Martins, [2]Gabriela A. Soares, [2]Bárbara H. S. Miranda, [2]Diego Bagnis, [3]Erica G. Chaves, [1*]Maria de Fátima V. Marques

[1]Instituto de Macromoléculas Professora Eloisa Mano (IMA), Universidade Federal do Rio de Janeiro, Av. Horácio Macedo, 2030, CT-Bloco J, 21941-598, Rio de Janeiro, RJ, Brazil.

[2]ONINN Centro de Inovações, Avenida José Cândido da Silveira, 2000, Horto Florestal, 31035-536, Belo Horizonte, MG, Brazil.

[3]Centro de Pesquisas, Desenvolvimento e Inovação Leopoldo Américo Miguez de Mello (Cenpes, PETROBRAS), Av. Horácio Macedo, 950, 21941-915, Rio de Janeiro, RJ, Brazil.

Correspondence

Prof. Maria de Fátima Vieira Marques

E-mail: fmarques@ima.ufrj.br

Supporting Information

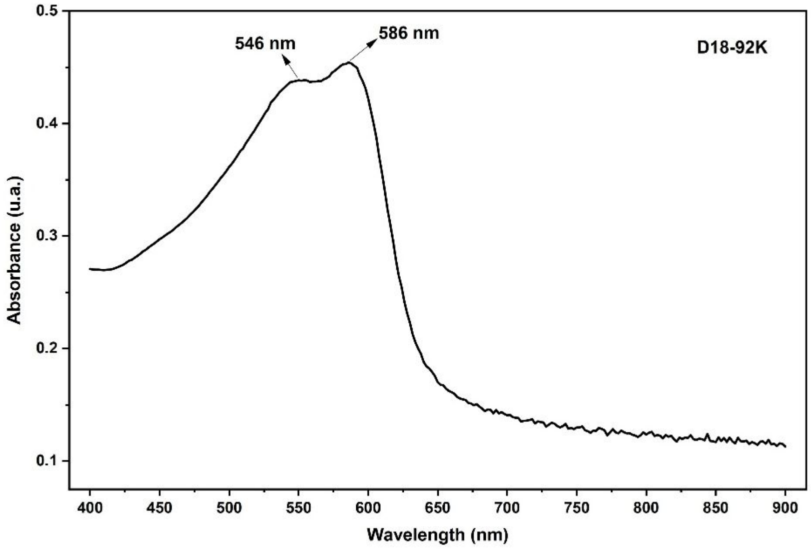

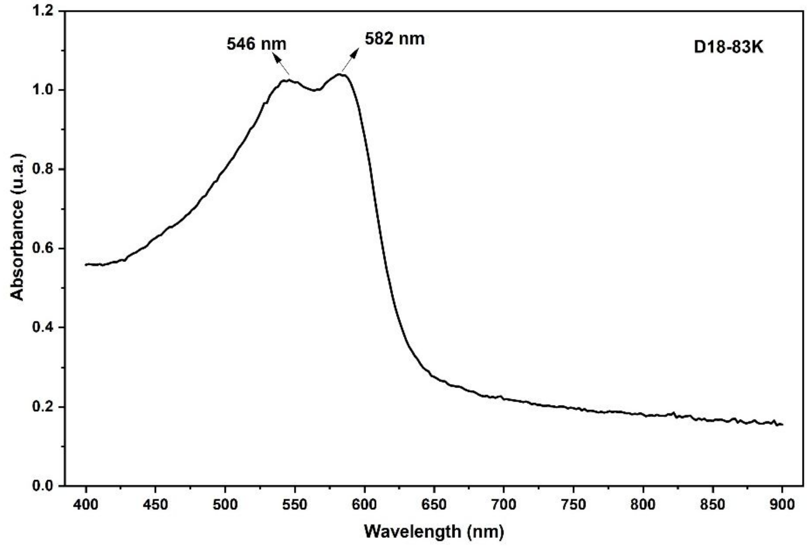

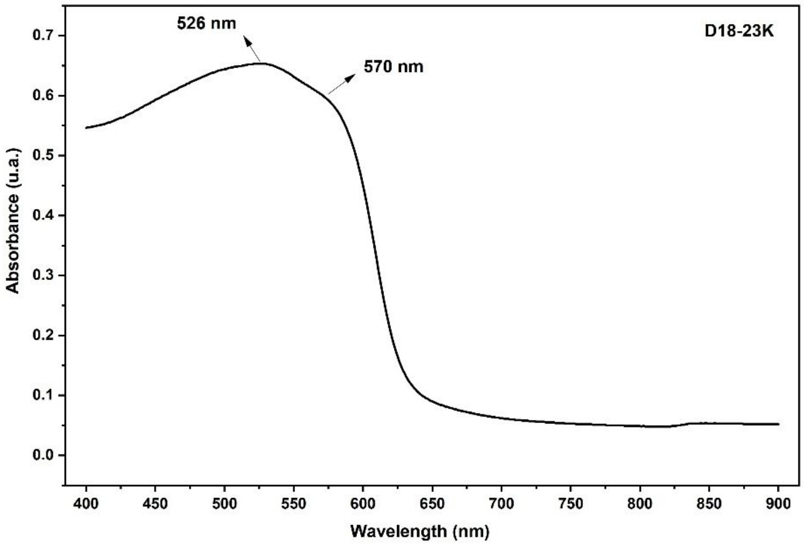
526 nm
570 nm
D18-23K
Absorbance (u.a.)
Wavelength (nm)

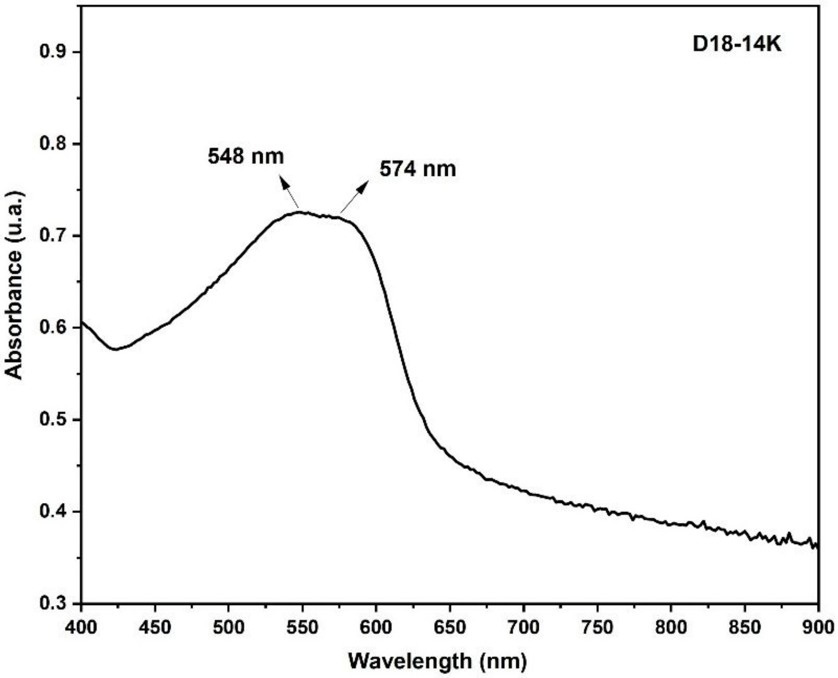
D18-14K
548 nm
574 nm
Absorbance (u.a.)
Wavelength (nm)

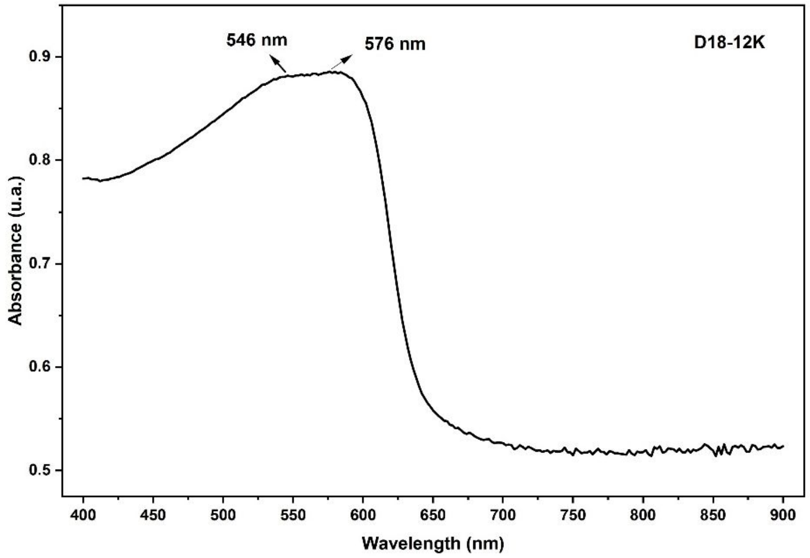

Figure S1. Thin-film UV–Vis absorption spectra of D18 polymers highlighting the vibronic features used for A_{0-0}/A_{0-1} ratio extraction.

Vibronic coupling analysis from UV–Vis spectra

A_{0-0} → lowest energy peak (largest λ)

A_{0-1} → adjacent highest energy peak (smallest λ)

Table S1. Visual estimation of wavelengths:

Polymer	λ_{0-0} (nm)	A_{0-0}	λ_{0-1} (nm)	A_{0-1}	(A_{0-0}/A_{0-1}) ratio
D18-12K	576	1.00	546	0.989	1.01
D18-14K	574	0.985	548	1.000	0.99
D18-23K	570	0.916	526	1.000	0.92
D18-92K	586	1.00	546	0.950	1.05
D18-83K	582	1.00	546	0.983	1.02
